# The Midwife Difficulty in New York

**Published:** 1898-03-05

**Authors:** 


### THE MIDWIFE DIFFICULTY IN NEW YORK.

It is clear that the "midwife difficulty" is by no means confined to England. In some parts of the United States the practice of midwifery is under more or less strict regulations, but we gather from a paper in the *Medical Record*, by Dr. Garrigues, that in New York City there is no law whatever regulating it. There, as here, great complaints are made as to the evils for which midwives are responsible. "Midwives not only do harm through their lack of obstetric knowledge, but they are most inveterate quacks. They treat, first of all, disturbances during the puerpery, later gynecologic diseases, then diseases of children, and are finally consulted in regard to almost everything. They never acknowledge their ignorance, and are always willing to give some advice. They administer potent drugs, such as ergot and opium. Their scarcely veiled advertisements in the newspapers show them to be willing abortionists; and since they have the right to give certificates of still-birth, who knows whether or not an infant's death is a natural one?" We hope we are not yet quite so bad as this in England, but if this is what the unrestricted practice of midwifery by unqualified and unregulated people leads to, the argument is very strong in favour of something being done. But what? Shall we end or shall we amend? Clearly the existing state of affairs, with all its possibilities, is intolerable.

